# Quarterly Report (No. 4,) of the Medical Diseases Affecting Children, Treated at the Westminster Station of the Royal Infirmary for Sick Children, from June 10th to September 10th, 1823

**Published:** 1823-10

**Authors:** John Webster


### STATISTICAL MEDICINE.

ART. I.—*Quarterly Report (No. 4,) of the Medical Diseases affecting Children, treated at the Westminster Station of the Royal Infirmary for Sick Children, from June* 10*th to September* 10*th*, 1823. By JOHN WEBSTER, M.D. &c.

*Diseases affecting particular Organs.*

| | | | | |
|---|---|---|---|---|
| Head and Nervous System | | Hydrocephalus Acutus | 2 | |
| | | Epilepsia | 1 | |
| | | Paralysis | 1 | |
| | | Hysteria | 1 | |
| | | Chorea Sancti Viti | 1 | = 6 |
| Organs of Respiration | | Tracheitis | 2 | |
| | | Bronchitis | 13 | |
| | | Pneumonia | 4 | |
| | | Phthisis | 2 | |
| | | Pertussis | 10 | = 31 |
| Organs of Digestion | Mouth, &c. | Aphtha | 6 | |
| | | Cynanche Parotidea | 8 | |
| | | ——— Tonsillaris | 5 | |
| | Stomach | Dyspepsia | 16 | |
| | Bowels | Constipatio | 13 | |
| | | Diarrhœa | 17 | |
| | | Dysenteria | 13 | |
| | | Enteritis | 1 | |
| | General | Cholera Morbus | 25 | |
| | | Marasmus | 3 | |
| | Worms | Lumbricus | 1 | |
| | | Ascarides | 17 | =119 |
| Organs of Generation | Uterus | Amœnorrhœa | 1 | |
| | | Menorrhagia | 1 | = 2 |
| Skin Eruptions | Acute | Variola Confluens | 11 | |
| | | Variola Discreta | 20 | |
| | | Variola post Vaccinam | 1 | |
| | | Varicella | 6 | |
| | | Morbilli | 11 | |
| | | Rubeola | 1 | |
| | | Milliaria | 2 | |
| | | Urticaria | 1 | |
| | | Scarlatina | 24 | |
| | Chronic | Psoriasis | 2 | = 78 |

*Diseases affecting various Parts.*

| | | | |
|---|---|---|---|
| Fevers | Continua Simplex | 9 | |
| | ——— Cephalica | 20 | |
| | ——— Pleuritica | 2 | |
| | ——— Gastro-enteritica | 42 | |
| | Remittens | 37 | |
| | Intermittens Quotidiana | 2 | |
| | ——— Tertiana | 1 | =113 |
| Muscles, Joints, &c. | Rheumatismus Acutus | 2 | = 2 |
| Cachexia | | 1 | |
| Vaccinated | | 49 | = 50 |
| | Total number of patients | | 401 |

*Fatal Cases reported at the Infirmary:*—Cholera Morbus, 3; Dysenteria, 1; Aphtha, 1; Marasmus, 1; Variola Confluens, 2; Phthisis, 1; Bronchitis, 1; Pneumonia, 1.—Total, 11.

During the three months embraced in the present Report, the most prevailing diseases have been those affecting the organs of digestion and the skin: of the former, many were exceedingly severe, and six cases are reported as having proved fatal; of the latter, small-pox and scarlatina were the most frequently observed, but amongst them only two instances of death are recorded.

Towards the end of the month of July, Cynanche was prevalent, and the six cases of Mumps stated in the table of diseases all occurred within the space of about a week,—a circumstance consonant with the observation of others; for at that time affections of a similar nature were very common. During the hot weather at the end of August and the beginning of September, Cholera Morbus was the most frequent and violent disease amongst the children at the Infirmary; and it is worth noticing that more fatal cases were then met with than during any similar period. Calomel, opium, and castor-oil, were found to be the most effectual remedies, and upon them the chief reliance for a cure was placed, especially in the early stages of the complaint; but, if the disease had existed for two or three days before application at the institution, medicine was of very doubtful efficacy, and the little patient sometimes sunk in spite of any effort made to save it.

Whilst remarking the unusual frequency of Small-pox, it may not be uninteresting to mention that a greater number of parents have applied to have their children vaccinated during the last three months than in any former quarter: thus proving that the lower orders not only continue to have faith in the efficacy of vaccination as a protection against small-pox, but they are even now more inclined to adopt that practice than formerly, although instances of failure do occasionally happen; of which one example is stated in the above report. In this case the disease was mild, but furnishes nothing particular, further than the child had been *properly* vaccinated some years before.

Of the fatal cases examined, the two following are thought worthy of being detailed, on account of the interesting pathological appearances met with. At both dissections Dr. LEE was also present; and it is principally from his pen that the subjoined minute history of the phenomena observed is given.

The subject of the first was two years and four months old, but never had enjoyed good health, nor been able, at any period of its life, to support the head. This child had been treated, at the Infirmary, for hooping-cough of considerable duration and severity, some months previous to the present application: this complaint, however, rapidly gave way to the exhibition of the remedies commonly prescribed,—namely, leeches to the forehead and purgatives. The patient was also at that time labouring under an enlargement of the spleen, which had been overlooked by the parents. A few months afterwards, this disease increasing, and being also accompanied with an affection of the organs of respiration, it was replaced on the register of the Infirmary; but medicine was of little avail, and the patient died after a lingering illness. Some weeks previous to death, when the spleen almost filled the abdomen, and the body was exceedingly emaciated, it may be remarked that the child had a constant craving for food, which it devoured in large quantities; and the stools were always of a whitish colour. The head was of an uncommon size and weight, and resembled very much that of an adult, the fontanelles being completely ossified. On sawing through the skull, the bones were found of an extraordinary thickness; in most places fully a fourth of an inch in diameter, and consisting of two tables and a diploe. The dura mater was divided at the same time, and the upper part removed with the bones. The arachnoid membrane and pia mater were thick and opaque, and in many parts covered with red patches of various sizes, resembling very strongly the petechial blotches occasionally seen on the surface of the body; and the whole upper surface was covered with a gelatinous-like exudation. On the inside of the dura mater, particularly where it lined the left parietal bone, there was a distinct adventitious membrane, very thin, and easily removable. The dura mater was also covered with red patches, similar to those on the pia mater. The convolutions of the brain were more fully developed than is generally seen in subjects so young; about six ounces of serum were found in the ventricles; the fourth was much distended, and the communication between it and the spinal cord was very large. There were adhesions between the pleura on both sides of the thorax; and the middle and inferior lobes of the right lung were hepatized: the left inferior lobe was in the same condition. The pleura on both sides was also covered with red patches of various sizes, resembling those found in the membranes of the brain. The other thoracic viscera were sound. In the larynx, the vocal cords were imbedded in a gelatinous matter, and a small ulcerated spot was observed under the right side of the thyroid cartilage. The spleen was about six times the natural size, of a darker colour and harder than usual. The other abdominal viscera were apparently sound.

The complete ossification of the cranium in this case, and the formation of an adventitious membrane on the internal surface of the dura mater, are two very uncommon circumstances, and rarely met with. Authors have certainly taken notice of similar appearances; and amongst these there can be no doubt that, in the following passage from Morgagni's viith Epistle, No. 15, the same state of the dura and pia mater is described: "Crassa meninx sublata interiorem faciem ostendit crebris coccineis maculis, quasi guttis sanguinis distinctam."

The other case was that of a girl, four years old, who, previous to an attack of small-pox six months ago, had enjoyed, according to the parent's account, good health. After the disappearance of the eruption, she was seized with pneumonic inflammation, and subsequently with disease of the mesenteric and cervical glands. Those of the neck on both sides were very much enlarged, and an indurated mass was distinctly felt through the abdominal parietes. For several weeks before death, she had suffered from hectic paroxysms and a gradual wasting; and, besides, she had had measles about a month previous to that event. On dissection, the ventricles of the brain were found to contain four ounces of fluid. The pia mater, covering the cerebellum and tuber annulare, was red, thickened, and opaque; and that over the middle lobes, near the fissura sylvii, was studded with small yellow-like specks; and the walls of the lateral ventricles were soft and pulpy. On opening the thorax, the pericardium was found to be greatly distended, and to compress the lungs around, to which it had formed adhesions: on opening it, a fluid escaped in a full stream, in consequence of which the quantity could not be ascertained. The whole exterior surface of the heart, and the interior of the pericardium, were covered with a thick, white, flocculent membrane: near the apex of the heart it had a shaggy, or even hairy, appearance; but this was less so near the basis. No adhesions existed between the heart and pericardium: the latter was thickened and inflamed, and could be separated with great facility into its two component membranes. Several of the bronchial glands, near the bifurcation of the trachea, was much enlarged, and had united so as to form a considerable mass, in the centre of which was a black, very fetid, ropy matter, resembling in all respects the sphacelated cellular substance seen in cases of carbuncle. These glands had formed adhesions with the pleura, as it is reflected from the heart, and were in immediate contact with that organ; and an opening was also found between this cavity containing the putrid matter and the left bronchial tubes. Tubercles were observed in the right lung; and, in various parts, the lungs had undergone that change which has been termed by M. LAENNEC "tuberculous infiltration. The mesenteric glands were much enlarged, indurated, and many of them filled with a yellow caseous-looking matter; and spots of inflammation were observed on the intestines, particularly the ileum. The other viscera were of a healthy appearance.

---

